# Intestinal infection following aerosol challenge of calves with *Mycobacterium avium *subspecies *paratuberculosis*

**DOI:** 10.1186/1297-9716-42-117

**Published:** 2011-12-03

**Authors:** Susanne WF Eisenberg, Ad P Koets, Mirjam Nielen, Dick Heederik, Rienske Mortier, Jeroen De Buck, Karin Orsel

**Affiliations:** 1 Department of Farm Animal Health, Faculty of Veterinary Medicine, Utrecht University, Yalelaan, Utrecht, The Netherlands; 2 Institute for Risk Assessment Sciences, Utrecht University, Jenalaan, Utrecht, The Netherlands; 3 Department of Production Animal Health, Faculty of Veterinary Medicine, University of Calgary, Hospital Dr NW, Calgary, Canada

## Abstract

A challenge experiment was performed to investigate whether administration of *Mycobacterium avium *subsp. *paratuberculosis *(MAP) via the respiratory route leads to MAP infection in calves. Eighteen calves from test negative dams were randomly allocated to four groups. Six calves were challenged with MAP nasally and six calves were challenged by transtracheal injection; three orally challenged calves served as positive controls, and three non challenged calves as negative controls. The challenge was performed as a nine-fold trickle dose, 10^7 ^CFU in total. Blood and faecal samples were collected frequently. Calves were euthanized three months post-challenge and extensively sampled. Blood samples were tested for the presence of antibodies and interferon gamma producing cells by ELISA. Faecal and tissue samples were cultured in a liquid culture system and the presence of MAP was confirmed by IS900 realtime PCR. Fourteen out of fifteen calves had no MAP antibody response. The negative controls remained negative; all positive controls became infected. Two nasally challenged calves showed a Purified Protein Derivative Avian (PPDA) specific interferon gamma response. In all nasally challenged calves, MAP positive intestinal samples were detected. In three calves of the nasal group MAP positive retropharyngeal lymph nodes or tonsils were detected. In all calves of the transtracheal group MAP positive intestinal tissues were detected as well and three had a MAP positive tracheobronchial lymph node. These findings indicate that inhalation of MAP aerosols can result in infection. These experimental results may be relevant for transmission under field conditions since viable MAP has been detected in dust on commercial dairy farms.

## Introduction

Paratuberculosis or Johne's disease (JD) is a chronic enteritis of ruminants caused by *Mycobacterium avium *subspecies *paratuberculosis *(MAP). The well-accepted transmission route of MAP is the oral uptake of bacteria by susceptible calves via colostrum, milk, water or food contaminated with faeces from MAP-shedding cattle [1]. In addition, intrauterine transmission has been described [2]. Due to limited effectiveness of control programs and the fact that eradication could not be achieved, other non-identified transmission routes have been suggested [3-5].

Recently, MAP has been identified in settled dust samples collected on dairy farms under experimental and field conditions [6,7]. Currently, JD prevention programs do not include management practices to reduce dust production, spread of dust or exposure of susceptible calves to dust. All existing recommendations to decrease the risk of new infections of MAP in dairy operations are directly aimed at reducing the infection rate in calves by decreasing the contact with faeces of adult cows [8,9]. MAP containing dust may cause infection in susceptible calves via ingestion due to normal calf behaviour (exploring the environment by licking and suckling). In addition, inhalation of MAP containing dust may also represent a route of transmission. In sheep, experimental intratracheal infection has been successful in the past [10]. However, inhalation of MAP by cattle has only been hypothesized as a possible route of transmission [4].

The current study was designed as a proof of principle experiment to investigate whether inhalation of MAP can establish infection in dairy calves. Particle size of dust determines how deep particles can penetrate into the lungs. In humans it has been determined that inhaled particles of approximately 5 μm will be cleared by the mucociliary system of the respiratory tract and subsequently ingested (inhalable dust). Most particles < 5 μm can reach the alveoli and are called respirable dust [11]. A similar deposition and clearing pattern of aerosols was found in the lungs of calves [12]. Therefore, the influence of particle size on clearing in the respiratory tract of calves was assumed to be similar to humans. Two inoculation routes were tested in this study, intratracheal inoculation mimicking the uptake of respirable dust and aerosolized MAP mimicking the uptake of inhalable dust particles. In early MAP infection (< 13 weeks) humoral immune responses as well as macroscopic and histological lesions are unlikely to occur [13,14]. After experimental MAP inoculation in calves, specific cellular immune responses could be detected early by interferon gamma assays and tissue culture could confirm infection status [15-17]. Therefore, colonization of tissue determined by culture was used to confirm successful intestinal infection in this study.

## Materials and methods

### Animals

Fourteen newborn Holstein Friesian male calves, one male twin and one male/female twin (total of 18) were obtained from nine dairy farms located around Calgary, Alberta, over a period of five weeks. Donor-herds were identified as low MAP prevalent (< 5%) in a MAP prevalence study in 2009 by testing individual faecal, serum and milk samples of cows over 36 months of age. Faecal samples were cultured by para-JEM automated MAP culturing (para-JEM^®^, TREK Diagnostic Systems, Cleveland, OH, USA) and serum and milk samples were analyzed for MAP antibodies by Pourquier ELISA (IDEXX Laboratories, Inc, Westbrook, Maine, USA). Only calves of first and second lactation cows were included in the study. Faecal and serum samples of dams were collected at the day of parturition to confirm individual animal negative MAP status by both liquid culture and ELISA. All samples of dams tested negative. Calves were separated from their dam directly after birth and a pre-colostral serum sample was collected and used to check for BVD carriers. The calves were transported to the research facility and fed 6 L of gamma-irradiated colostrum (Hamilton McMaster Nuclear Reactor, Ontario, Canada) within 6 h, followed by milk replacer and calf starter grain without antimicrobial additives.

Calves were housed individually on sawdust bedding in a biosecurity level 2 barn. The barn was heated and the temperature was maintained at a minimum of 14°C for the duration of the trial. The health status was monitored on a daily base by clinical inspection. The protocol was approved by the Health Sciences Animal Care Committee (M09083) at the University of Calgary and performed according to Canadian Council of Animal Care regulations.

### Inoculum

A virulent cattle type MAP strain isolated from a clinical JD case in Alberta (Cow 69), with an IS900-RFLP profile identical to the K10 reference strain recommended in the literature, was used [18]. To mimic field conditions the dosage chosen was close to one of the lowest described to be infective when administered orally [16] which was also plausible for uptake through respiration. Data about dust concentrations in cattle housing [19,20] and the tidal volume of calves (8 mL/kg bodyweight) were used to estimate that a calf approximately inhales 700 mg of dust in the first 3 months of life via respiration alone excluding exploratory licking and suckling behaviour. It was shown that 10^5 ^CFU of viable MAP/mg dust can be detected in dust samples on commercial dairy farms [7]. The challenge dose was 8 × 10^5 ^CFU/dose administered on 3 consecutive days for 3 weeks in a row (trickle dose) leading to exposure of calves to approximately 1 × 10^7 ^CFU which is one of the lowest dosages described to cause infection after oral inoculation [16].

MAP colonies were grown in 7H9/mycobactin/glycerol/OADC broth at 37°C shaking at 100 RPM. The inoculum was tested for contamination with Gram staining and subculture to blood agar. The inoculum was vortexed with 3-4 1 mm glass beads to eliminate clumps and checked for viability using a fluorescent Live/Dead^® ^BacLight™ Bacterial Viability kit (Invitrogen, Burlington, ON, Canada). Subsequently, MAP was quantified using the "pelleted wet weight method" as previously described, where the pelleted wet weight averaged approximately 1 × 10^7 ^CFU/mg [18]. Aliquots of 1.7 mg of MAP/tube were frozen at -80°C to be used as inoculum for each inoculation series. A separate aliquot of MAP was used for each week of inoculation.

Prior to each inoculation, a tube was thawed and inoculated in 100 mL of 7H9/mycobactin/glycerol/OADC medium at 37°C shaking at 100 RPM for 5 days. MAP dose per mL was assessed using an in-house quantitative realtime PCR with a standard curve based on the 16 s rRNA gene of *Mycobacterium smegmatis *confirming the presence and the quantity of the 16 s rRNA gene using primers p882 (5'-aggattagataccctggtag-3') and p1100 (5'-gctgacgacatccatgc-3'). The inoculum was diluted to achieve the desired concentration of 8 × 10^5 ^CFU/mL and was stored at 4°C until just prior to the inoculation for a maximum of three days.

### Study design

Calves were assigned randomly to one of the four groups. Six calves were inoculated by transtracheal inoculation, six by nasal aspiration of aerosolized MAP, three were inoculated orally, and three did not receive a MAP challenge and served as negative control. Transtracheal inoculation with 5 mL of inoculum was performed using a technique described in the literature for the collection of bronchoalveolar fluid in calves without sedation [21]. Aerosolized inoculums (5 mL) with a variable size were administered using a nasal spray pump during inspiration of the calf directly into the nostril [22]. Oral inoculation was performed to prove pathogenicity of the administered strain at the dosage used for the other challenge routes by allowing calves to suckle a syringe containing 5 mL of MAP suspension. Negative controls were used to support the negative status of the collected calves and to detect unexpected MAP transmission during the trial. In addition, dust samples were collected with an electrostatic dust collector (EDC) to detect environmental contamination with MAP.

An overview of all samples collected during the trial is given in Table 1. At week 12 after the first challenge, euthanasia was performed using intravenous injection of barbiturate (pentobarbital sodium 540 mg/mL, Euthanyl Forte^®^, DIN 00241326, Bimeda-MTC Animal Health, Cambridge, Ontario, Canada) and necropsy was performed immediately afterwards.

**Table 1:** Overview of samples collected during the trial.

Type of sample	Total	Frequency	Pool	Analysis	Reason
		F0; before inoculation	3 calves per pool: 1: oral 2: no challenge 3a&b: T-tracheal 4a&b: Nasal		Faecal MAP shedding before infection
Faeces	13 per calf	Week 1 F1-3 pi Week 2 F4-6 pi Week 3 F7-9 pi; Each of 3 days following weekly inoculation.	3 samples of 2 calves per pool: 1a&b: oral 2a&b: no challenge 3a, b&c: T-tracheal 4a, b&c: Nasal	Liquid MAP culture with confirmation IS900 PCR	Passive faecal MAP shedding after inoculation
		Week 6 F10 pi	3 calves per pool: 1: oral 2: no challenge 3a&b: T-tracheal 4a&b: Nasal		Faecal MAP Shedding
		Week 9 F11 pi	Individually		
		Week 12 F12 pi	Individually		Faecal MAP Shedding
Dust	18; at each stall	Week 11 pi	Individually	Liquid MAP culture with confirmation IS900 PCR	Environmental contamination with MAP
Serum	2 per calf	Week 0 pi Week 12 pi	Individually	Commercial JD-ELISA	B-cell activation& seroconversion
Whole blood	1 per calf	Week 12 pi	Individually	Bovigam^® ^interferon gamma essay	T-cell sensitisation
Tissue	18 (19*) per calf	W 12 pi	Individually	Liquid MAP culture with confirmation IS900 PCR	Tissue colonisation with MAP

Overview of the samples collected. Frequencies and time points of collection and tests used for analysis during the 12 week trial are described. Additionally, the reason for sampling is indicated. F = faecal sample, pi = post inoculation, T-tracheal = transtracheal. *an additional piece of lung was sampled from one calf.

### Necropsies

During necropsies no other ruminants were allowed in the necropsy room. One calf was opened at a time. At post-mortem, a total of 18 tissue samples were collected per calf with separate sterile instruments for each sample. Three parts of the ileum were sampled with adjacent lymph nodes (ileocaecal valve, middle and proximal parts of the ileum) as well as four parts of the jejunum with adjacent lymph nodes (0.5, 1, 1.5 and 2 m proximal from the proximal ileum). Additionally, the tonsils, the retropharyngeal lymph nodes and right cranioventral lobe of the lung with tracheobroncheal lymph nodes were collected. In case of macroscopic lung pathology the abnormal lung tissue was sampled additionally. To minimize cross contamination, samples were collected in the following order: tonsils, retropharyngeal lymph nodes and lung tissue were collected first, then parts of interest of the intestinal tract were identified, marked and adjacent lymph nodes were collected before opening and sampling the intestinal tissue. Equipment and the necropsy room were cleaned and disinfected between necropsies.

### Sample analysis

Serum samples of the calves were analysed for MAP specific antibodies using Pourquier ELISA following the manufacturer's instructions. The results were expressed as sample to positive ratio (S/P-ratio) using the kit positive control to correct for inter-plate variation as per the manufacturers instruction.

Whole blood was analyzed by Bovigam^® ^(Prionics, Lavista, USA) to detect T-cell sensitization. Samples were stimulated within 8 hours after blood collection in a 24-well tissue culture plate. Commercially available antigens were used to incubate cells. Purified Protein Derivative Avian (PPDA; Prionics) and Purified Protein Derivative Bovis (PPDB; Prionics) included with the Bovigam^® ^assay were used as specific antigens. Pokeweed mitogen (PMT; Sigma Aldrich, Oakville, Ontario) was used as a positive control and PBS (pH 7.4) as a negative control. The supernatant was stored at -20°C until analysis. The results were expressed as the S/N ratio using the PBS stimulated sample to correct as per the manufacturer's instruction.

Faecal samples of calves were analysed as described in Table 1 and dust samples were tested individually. Dust samples were decontaminated and prepared as described previously [7]. All faecal samples were decontaminated and prepared for culturing in liquid medium (TREK para-JEM^®^) according to the instructions provided by the manufacturer.

Intestinal samples (4 × 4 cm) were rinsed in PBS to remove intestinal contents and the mucosa was scraped off the intestinal wall using slides. The tissue was put into 50 mL tubes prefilled with 20 mL of 1/2 strength Brain Heart Infusion (BHI) containing 0.9% hexadecylpyridinium chloride (HPC). The lymph nodes (2 g) and pieces of lung were cut open and put into stomacher bags with 5 mL of NaCl. Samples were homogenized with a Stomacher^® ^(Stomacher^® ^80 Biomaster, Seward Laboratory Systems Inc., Bohemia, New York, United States) for 1 min at high speed. Fluid containing debris was poured into a 50 mL tube containing 20 mL 1/2 strength BHI with 0.9% HPC. Samples were incubated overnight at 35°C. The next day, tubes were centrifuged at 1700 *g *for 20 min, the supernatant was discarded, the pellet was resuspended in 1 mL 1/2 strength BHI containing antibiotics (vancomycin and naladixic acid 100 μg/mL, amphotericin 50 μg/mL) and incubated overnight.

After these initial decontamination and preparation steps faecal, dust and tissue samples were incubated at 35°C for 42 days in para-JEM^® ^liquid culture medium. MAP presence was confirmed by IS900 realtime PCR on culture medium as previously described [7]. Culture results were considered as a binominal outcome (MAP detected/not detected).

### Data analysis

Interferon gamma results were analyzed by ANOVA analysis comparing the S/N-ratios of the 4 experimental groups. The number of positive tissue cultures per calf was recorded and presented per location. An inoculation route was considered to be successful if at least one calf per group had at least one culture positive intestinal tissue.

## Results

### Clinical findings

During the study no severe clinical abnormalities were detected. Several calves had a dry cough throughout the experimental period. It occurred in all groups before and also several weeks after inoculation. One calf in the nasal group was diagnosed with and treated for pneumonia within the first week of life before inoculation. Slight diarrhoea was detected in 3 calves; in one it occurred before inoculation, and in the other 2 in week 6 post inoculation (pi).

Calves showed no reaction after nasal inoculation. Some calves inoculated trans-tracheally reacted with a slight cough to the injection of fluid into the trachea. Twice traces of blood were detected in the nostrils after transtracheal injection.

### Immune parameters

Serum samples of calves at day 3 of life and D82 after inoculation were negative for MAP specific antibodies in the ELISA with the exception of one calf from the oral inoculation group.

In all calves, interferon gamma production of cells stimulated with PBS and PPDB were negligible. Stimulation with PMT showed a clear interferon gamma response in all animals. Two calves of the nasal inoculation group had a more prominent interferon gamma response to PMT. After stimulation with PPDA the interferon gamma production was comparably low in the negative control, the positive control and the transtracheal inoculated group. In the nasal inoculation group, the two calves with the prominent interferon gamma response to PMT also showed a high response to PPDA. A significant (*p *= 0.002) difference was found between the nasal inoculated and the positive control group.

### Necropsy

No macroscopic intestinal lesions were observed in any of the calves at necropsy. The calf in the nasal group treated for pneumonia showed a few pleural adhesions. No MAP could be detected in an additional piece of lung tissue collected underneath the adhesions.

### Culture results

All faecal samples of the calves collected throughout the trial were culture negative. All 18 dust samples collected 8 weeks after the last inoculation were also culture negative. Culture results of tissue samples are summarized in Table 2. No MAP could be detected in the tissue of the negative control group. Intestinal MAP infection occurred in all inoculated calves.

**Table 2:** Summary of tissue culture results.

Group	Total no. Positive out of 18	CaeV	CaeV ln	Il 1	Il 1 ln	Il 2	Il 2 ln	Je 1	Je 1 ln	Je 2	Je 2 ln	Je 3	Je 3 ln	Je 4	Je 4 ln	Lu 1	TB	Re	To
No chal.	0																		
No chal.	0																		
No chal.	0																		

Oral	1										**+**								
Oral	2										**+**	**+**							
Oral	1	**+**																	

Nasal	7			**+**	**+**	**+**	**+**	**+**					**+**					**+**	
Nasal	1				**+**														
Nasal	1							**+**											
Nasal	3						**+**								**+**				**+**
Nasal	1						**+**												
Nasal	4					**+**							**+**		**+**			**+**	

T-Trach	4									**+**		**+**		**+**	**+**				
T-Trach	2							**+**									**+**		
T-Trach	2								**+**								**+**		
T-Trach	4				**+**			**+**	**+**								**+**		
T-Trach	3									**+**	**+**		**+**						
T-Trach	2											**+**		**+**					

MAP presence in tissue was confirmed by IS900 realtime PCR on liquid culture medium. No chal: no challenge, T-trach: transtracheal; CaeV = caecoilio valves, ln = lymph node, Il = ileum, Je = jejunum, Lu = lung, TB = tracheobroncheal lymph node, Re = retropharyngeal lymph node, To = tonsils.

Nasal inoculated calves showed up to 6 MAP positive intestinal tissue samples, whereas transtracheal inoculated calves had a maximum of 4 positive intestinal tissue samples. Nasal inoculation led to the presence of MAP in the tonsils of 1 calf and in the retropharyngeal lymph node in 2 other calves. Transtracheal application of MAP resulted in a positive tracheobronchial lymph node in 3 calves.

## Discussion

This study supports the hypothesis that MAP might be transmitted via bioaerosols since a low trickle dose administered via nasal and transtracheal routes could establish MAP infection in all inoculated calves. Both respiratory routes caused infection of intestinal tissue and intestinal lymph nodes.

In sheep intratracheal MAP exposure has been previously described as a potential route of infection [10]. The number of calves used in this study was too small to statistically compare results for tissue locations between groups, but the goal was a proof of principle. The presence of MAP in cultured intestinal tissue and lymph node samples of all challenged calves (Table 2) supports that MAP uptake via the respiratory tract can induce MAP infection in susceptible calves. Recent detection of MAP in bioaerosols on commercial dairy farms indicates that this route has to be considered as a possible route of transmission [6].

Intermittent faecal shedding due to infection has been described in experimental trials as early as 146 days post challenge [13]. Since calves in this study were inoculated with a lower dose per inoculation day compared to literature and were euthanized at 12 weeks pi, no presence of MAP in any faecal sample was expected. In early MAP infection, no macroscopic lesions occur and histological lesions containing acid-fast bacteria can be detected sporadically if at all [13,14]. Therefore in this study, confirmation of MAP infection in tissue samples was performed by MAP culture.

The detection of seroconversion against MAP using commercially available ELISA tests has occurred earlier in experimental infection studies than in naturally infected cattle, but not before 406 days after inoculation [13,23,24]. The absence of MAP specific antibodies shortly after birth showed the absence of maternal antibodies in colostrum administered to the calves. Not detecting antibodies in 14 out of 15 challenged calves in week 12 after inoculation was in agreement with published studies indicating seroconversion so early after challenge to be a rare event. The interferon gamma assay detects cellular immune responses and was deemed useful for early paratuberculosis detection in cattle [15,25]. In experimental studies in calves, interferon gamma production has been detected as early as day 7 after intra-peritoneal inoculation [15]. However, after oral inoculation (4 times of 1-2 × 10^10 ^CFU/dose) interferon gamma production did not increase before day 90 pi and was considered a robust measure only after 6 months of age [15]. In the present study, calves were inoculated with a considerably lower dose and were euthanised before 90 days post inoculation (dpi) explaining the low specific interferon gamma response in the oral inoculation group. Surprisingly, two calves inoculated through the nasal route showed a clear interferon gamma response after specific antigen stimulation as early as 12 weeks pi. These findings and negligible responses to stimulation with PBS and PPDB indicate a specific sensitization of peripheral blood mononuclear cells to MAP after nasal inoculation.

Faecal-oral transmission of MAP is believed to be the most common route of transmission under field conditions [1,26]. In addition, this route of inoculation has been frequently confirmed to be effective in experiments [18]. Therefore, the positive control group was inoculated orally with the same inoculation scheme and dose as the experimental groups. All 3 positive control calves became infected (Table 2), although the total dose (1 × 10^7 ^CFU/dose) and the dose given per inoculation day (8 × 10^5 ^CFU/dose) was low [18]. A dose of 1.5 × 10^6 ^CFU/dose for 2 consecutive days reliably induced detectable infection, whereas a dose of 2 × 10^5 ^CFU/dose did not [16]. In this study the daily oral dosage was lower than 10^6 ^CFU/dose and probably only led to an infection because it was administered several times as a trickle dose adding up to a total dose around 10^7 ^CFU.

In humans, aerosol routing after aspiration has been described to be dependent on droplet size [11,27]. Similar estimates for particle penetration into the respiratory tract have been performed in calves [12]. To mimic aerosol infection by droplets in the upper respiratory tract a device was used to administer the inoculum to the nasal group which was designed for intranasal application in humans. It produced aerosols with a variable size which were mainly trapped in the mucociliary system of the nose [22]. Thus, most MAP would have reached only the nasal cavity passing the nasal associated lymphoid tissue (NALT) before being cleared by the mucociliary system and ingested [28]. NALT contains M-cells which have been shown to facilitate MAP uptake in Peyers patches of gut associated lymphoid tissue (GALT) after oral uptake [29,30]. After nasal inoculation, MAP would come into direct contact with NALT and thus with M cells; it would also pass by the palatine tonsils before being swallowed and diluted by saliva. Therefore, when presented to M cells in NALT, uptake of MAP might occur via a similar mechanism to that which occurs during oral infection; respiratory uptake may be even more efficient since in this case MAP is not diluted in intestinal contents before reaching the target tissue. In addition, when inhaled, surplus MAP will be swallowed and M-cells in the Peyers patches subsequently will be exposed. Confirmation of MAP in retropharyngeal lymph nodes and in tonsils of calves of the nasal inoculation group supports that the nose can act as a portal of entry for MAP.

Transtracheal application of MAP was performed, mimicking the scenario of MAP attached to respirable dust. Particles and bacteria entering alveoli will be taken up by pulmonary-alveolar macrophages and removed by the mucociliary system, passing the tonsils before being swallowed; alternatively they could pass the mucosal epithelial barrier [12,31,32]. M-cells in bronchus-associated lymphoid tissue (BALT) have been described as a portal of entry for *Mycobacterium bovis *in mice [33]. BALT is not present in the neonatal bovine lung, but can be detected after 4 months of age [34] indicating that BALT could play a role in MAP uptake in older young stock but not in the present study. In this study, MAP was possibly cleared by pulmonary-alveolar macrophages and partially passed the mucosal epithelial barrier indicated by confirmed positive tracheobroncheal lymph nodes in 3 calves of the transtracheal group. These findings support that the lung can act as a portal of entry for MAP.

After oral inoculation and uptake by M cells of the Peyers patches, macrophages are the target cells since MAP is capable of interfering with bactericidal mechanisms of these cells [35]. Within macrophages, MAP migrates to the regional lymph nodes [14] and in later stages of disease the whole body is colonized [36-38]. Older oral infection studies detected MAP in lymphoid tissue of the pharyngeal area shortly after inoculation, followed later on by the intestinal tract and other organs indicating dissemination via the reticuloendothelial system to reach the intestinal tissue [39,40]. This theory was supported by the finding that instillation of MAP in the tonsils of calves' leads to infected intestinal tissue [13]. In addition, intraperitoneal inoculation proved to be effective for inducing infection [41]. In this study MAP lymph nodes close to the inoculation site were positive, as well as intestinal tissue and lymph nodes, indicating that migration occurred as well. Intestinal colonization when inoculated orally, intraperitoneally and directly into the tonsils and now also nasally and transtracheally supports that migration of MAP has to occur once it has passed the epithelial barrier [16,41,42].

In conclusion, intestinal MAP infection can be induced via the respiratory route when administered into the nose or the trachea of young calves. If MAP uptake after inoculation occurred through the nose and lung tissue directly as hypothesized or due to ingestion after mucociliary clearance maintains unclear. Nevertheless intestinal infection occurred indicating that bioaerosols can be a route of transmission. Since MAP has been detected in bioaerosols on dairy farms, the transmission of MAP by bioaerosols will need to be considered in MAP control programs in addition to the faecal-oral route.

## Competing interests

The authors declare that they have no competing interests.

## Authors' contributions

SFWE designed and conducted the experiment, collected and analysed the samples and wrote the draft manuscript. APK designed the experiment, developed the inoculation procedure and helped to draft the manuscript. MN designed the study and helped to draft the manuscript. DH designed the study and helped to draft the manuscript. RM was involved in animal logistics, took care of inoculum preparation and analysed blood samples. JDB developed animal logistics and helped to draft the manuscript. KO designed and supervised the experiment and helped to draft the manuscript. All authors read and approved the final manuscript.
